# Supplementary figures and images for: CRISPR screening identifies M1AP as a new MYC regulator with a promoter-reporter system
Source: PeerJ. 2020 May 6;8:e9046. doi: 10.7717/peerj.9046 (PMC7210806; doi:10.7717/peerj.9046)

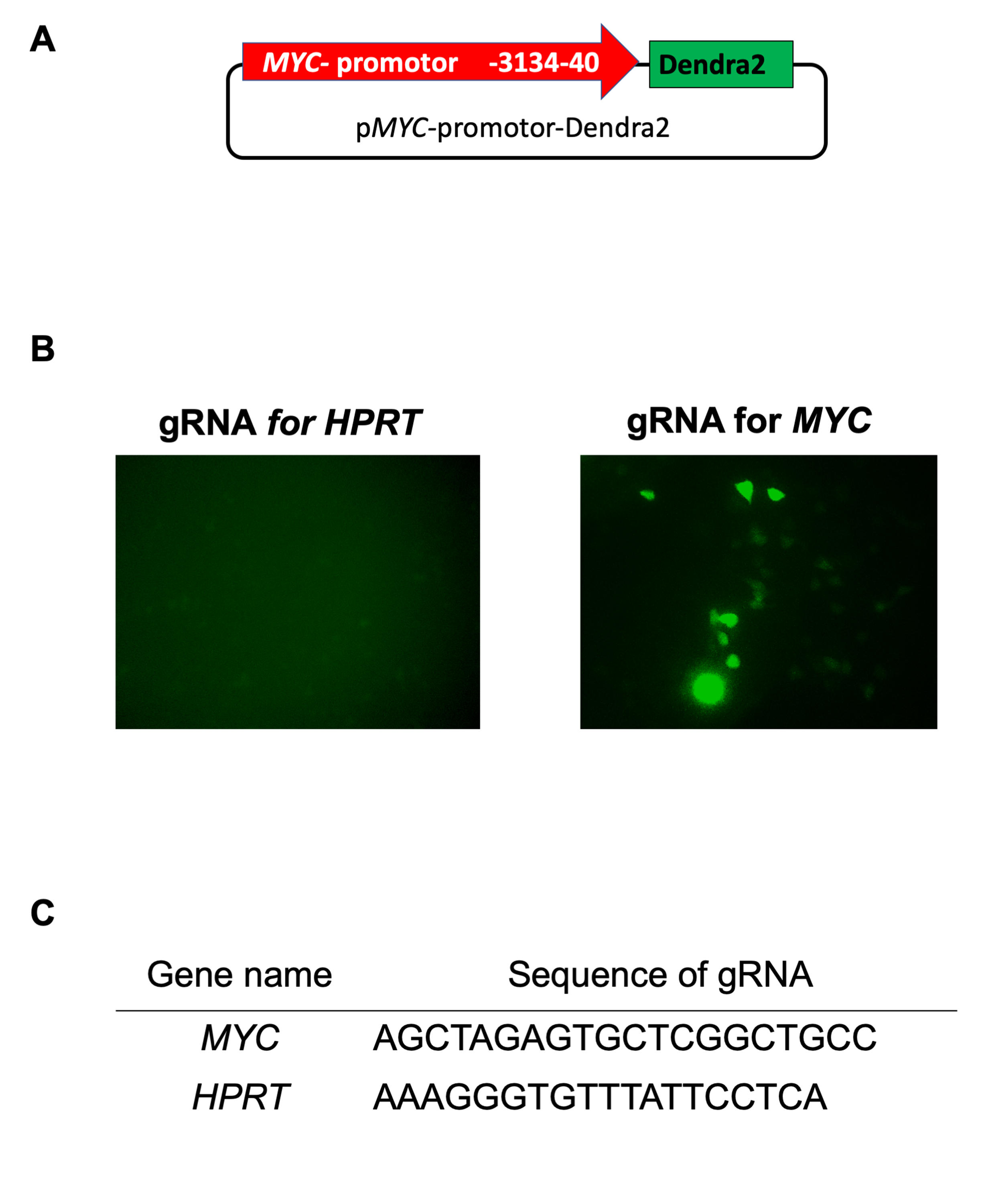

Supplement: Figure S1 [file peerj-08-9046-s001.png]

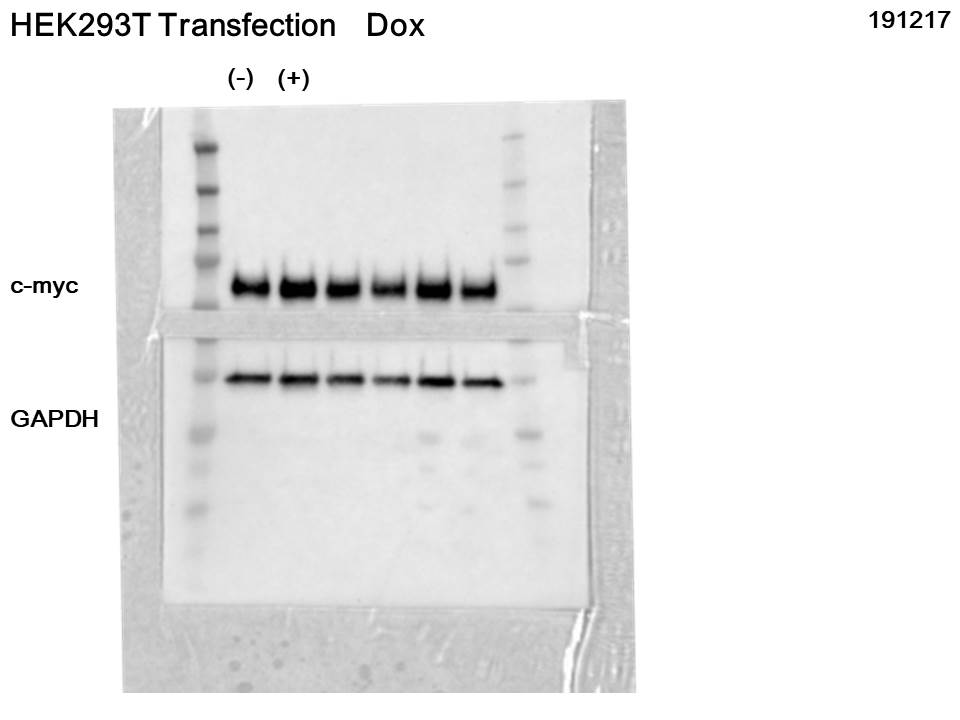

Supplement: Supplemental Information 2 [file peerj-08-9046-s002.jpg]

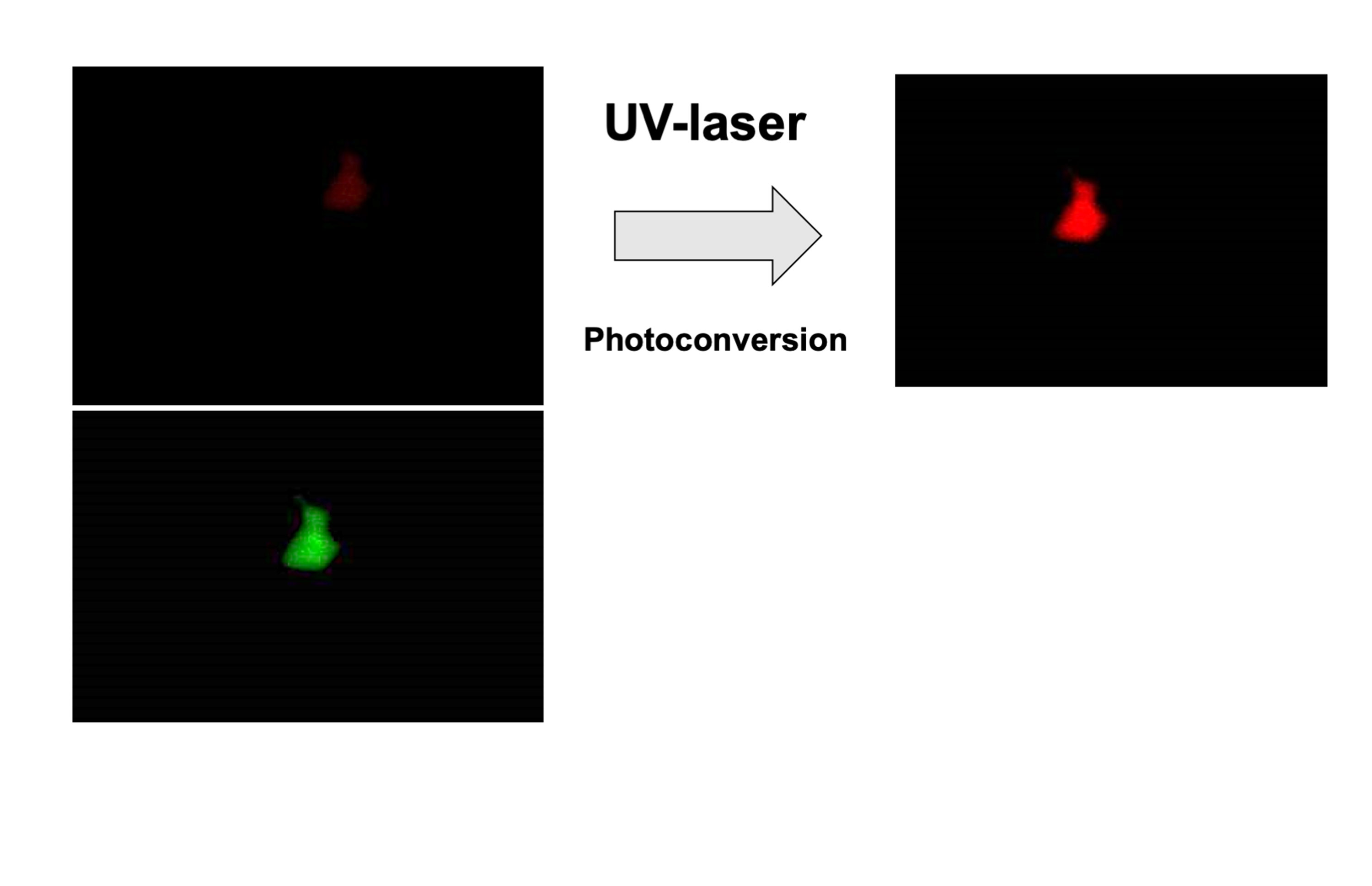

Supplement: Figure S2 [file peerj-08-9046-s003.png]

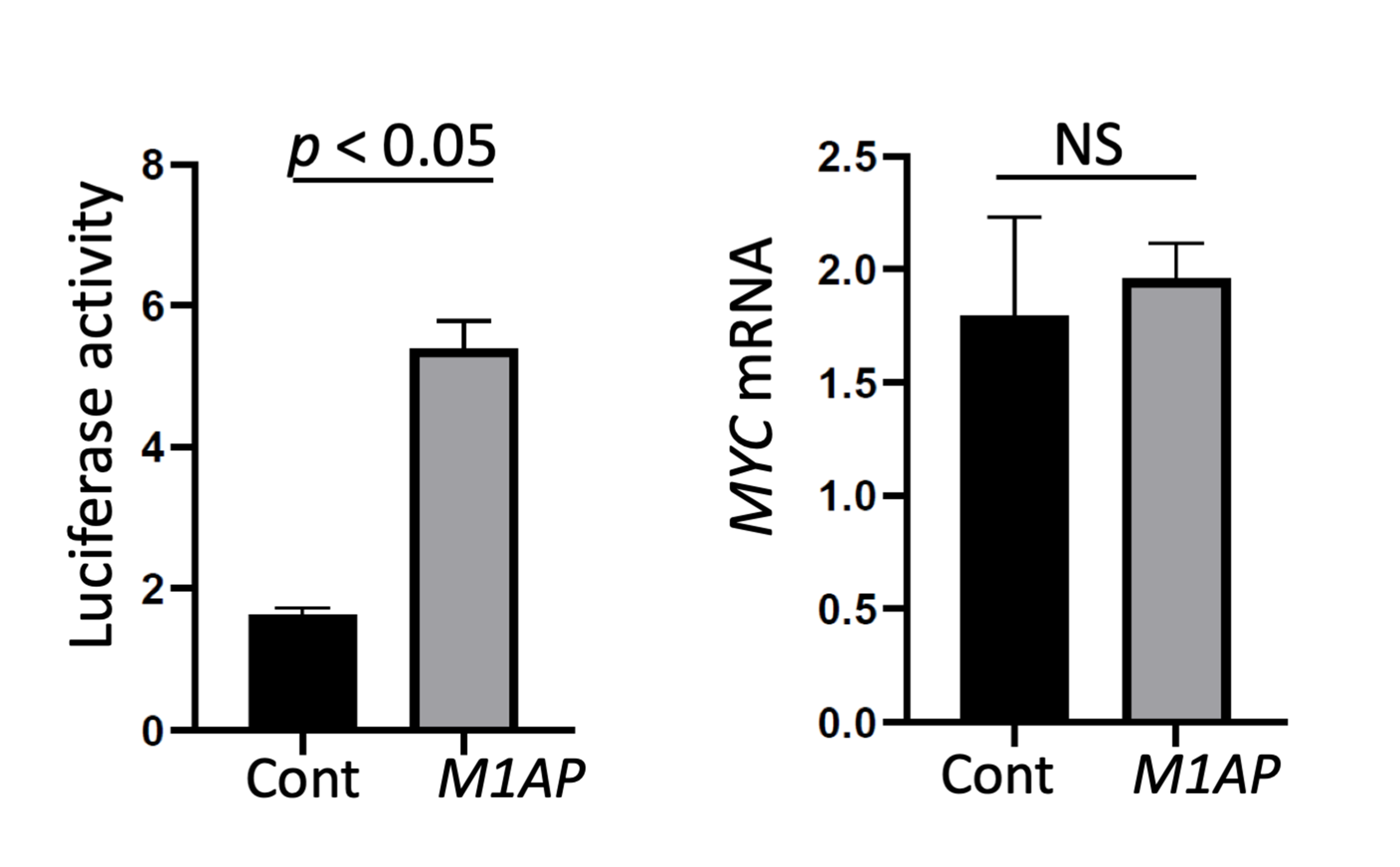

Supplement: Figure S3 — MYC promoters are strongly activated at 72 h after induction of M1AP expression, but the mRNA of MYC was not significantly upregulated at 72 h. [file peerj-08-9046-s004.png]

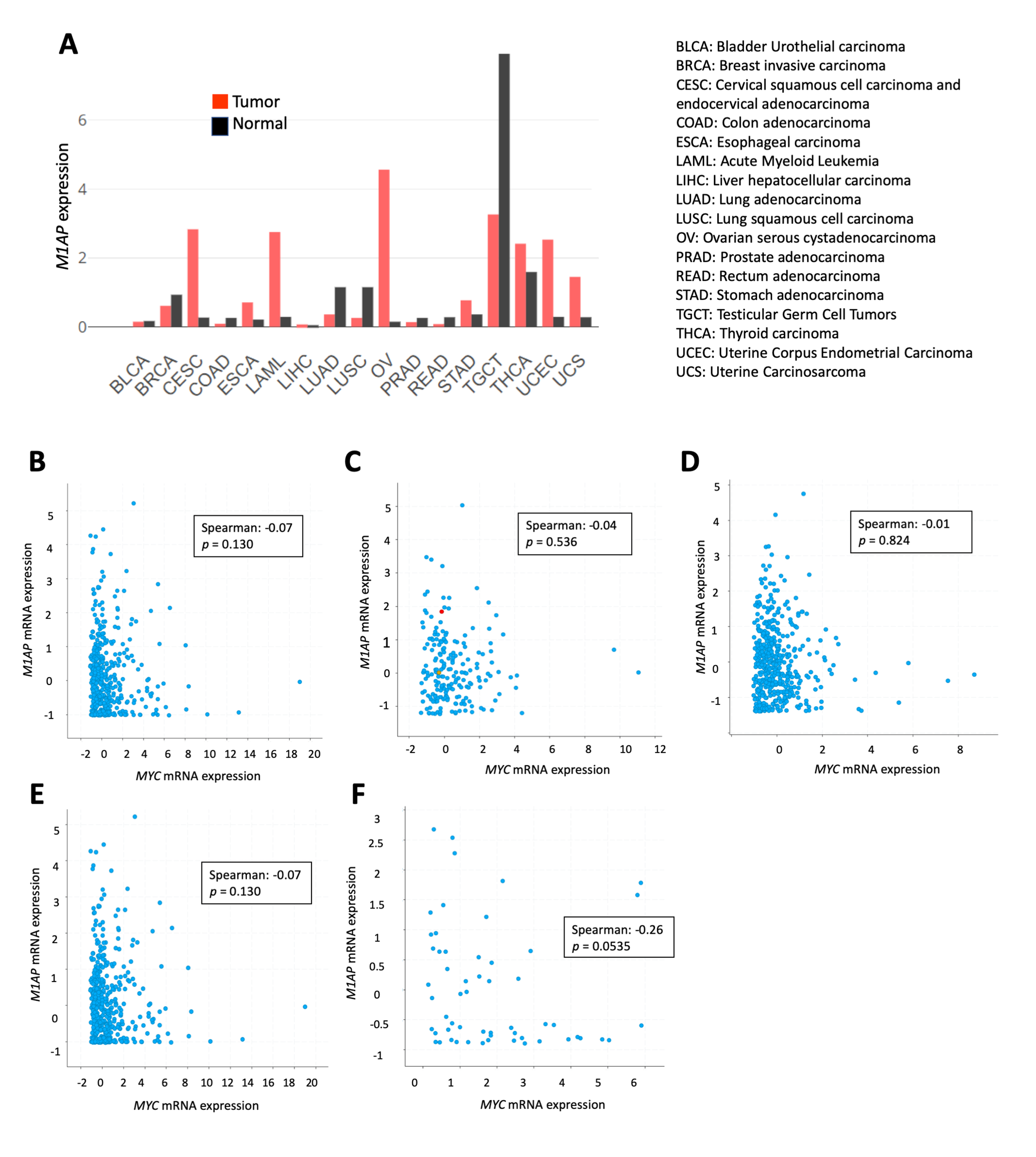

Supplement: Figure S4 — (A) Comparison of M1AP expression in normal and tumor tissue in GEPIA. (B)–(F) Correlations between MYC and M1AP in cBioPortal. (B) Cervical carcinoma. (C) Ovarian carcinoma. (D) Thyroid carcinoma. (E) Uterine endometrial carcinoma. (F) Uterine carcinosarcoma. [file peerj-08-9046-s005.png]
