# Supplementary material for: CRISPR screening identifies M1AP as a new MYC regulator with a promoter-reporter system
Source: PeerJ. 2020 May 6;8:e9046. doi: 10.7717/peerj.9046 (PMC7210806; doi:10.7717/peerj.9046)
Supplement: Supplemental Information 6 [file peerj-08-9046-s006.pdf]

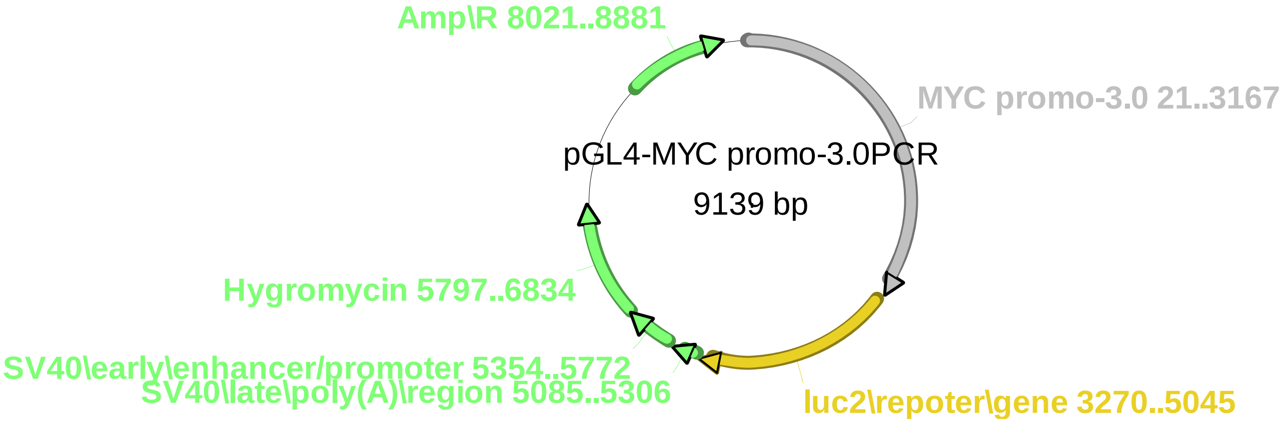

Amp\R 8021..8881
MYC promo-3.0 21..3167
pGL4-MYC promo-3.0PCR
9139 bp
Hygromycin 5797..6834
SV40\early\enhancer/promoter 5354..5772
SV40\late\poly(A)\region 5085..5306
luc2\repoter\gene 3270..5045
